# Langerhans cell histiocytosis in children with refractory diarrhoea and hypoalbuminaemia as the initial presentation: two case reports and a literature review

**DOI:** 10.1186/s12887-024-04612-6

**Published:** 2024-03-22

**Authors:** Yi Cao, Qing-Qing Wu, Wei-Hui Yan, Li-Na Lu, Yi-Jing Tao, Hai-Xia Feng, Yi-Jing Chu, Wei Cai, Ying Wang

**Affiliations:** 1 Division of Pediatric Gastroenterology and Nutrition, Xinhua Hospital Affiliated to Shanghai Jiao Tong University School of Medicine, No. 1665, Kongjiang Rd, Shanghai, China; 2 Shanghai Institute for Pediatric Research, Shanghai, China; 3 Shanghai Key Laboratory of Pediatric Gastroenterology and Nutrition, Shanghai, China

**Keywords:** Langerhans cell histiocytosis, Gastrointestinal tract, Endoscopy, Rash, PET-CT

## Abstract

Langerhans cell histiocytosis (LCH) involving the gastrointestinal tract is a rare condition for which clinical experience is limited. We describe the cases of two patients who initially presented with chronic diarrhoea, hypoproteinaemia, and intermittent fever. These findings suggest that in cases of refractory diarrhoea accompanied by recurrent hypoalbuminaemia, especially with abdominal rash, LCH should be considered. Gastrointestinal endoscopy, biopsy, and imaging studies are essential for obtaining a definitive diagnosis. This approach might be helpful for the early recognition of gastrointestinal tract involvement in LCH.

## Introduction

Langerhans cell histiocytosis (LCH) is a histiocytic disorder characterized by the abnormal proliferation and dissemination of Langerhans cells derived from the bone marrow [1]. Although LCH has a wide clinical spectrum, gastrointestinal tract (GIT) involvement in LCH is extremely rare, and its manifestations are variable and nonspecific. We report that two patients less than 2 years old presented with chronic diarrhoea, recurrent hypoalbuminaemia, abdominal rash and intermittent fever. Unexplained diarrhoea lasting longer than 2 months was considered refractory diarrhoea. The serum ALB concentration was lower than 35 g/L at admission and rebounded transiently with albumin supplementation and then decreased significantly a few days later, indicating recurrent hypoalbuminaemia. The purpose of this paper is to improve clinicians’ understanding of LCH.

## Case presentation

### Patient 1

An 18-month-old girl was admitted to the hospital with a 5-month history of diarrhoea (intermittently mixed with blood) and generalized anasarca and intermittent fever for more than 1 month. She was admitted to a local hospital 3 months ago and her caregiver was instructed to change her formula to amino acid-based formula, but there was no improvement in her diarrhoea over the course of 3 months. As a result, she was transferred to our hospital for treatment.

**Fig. 1:**
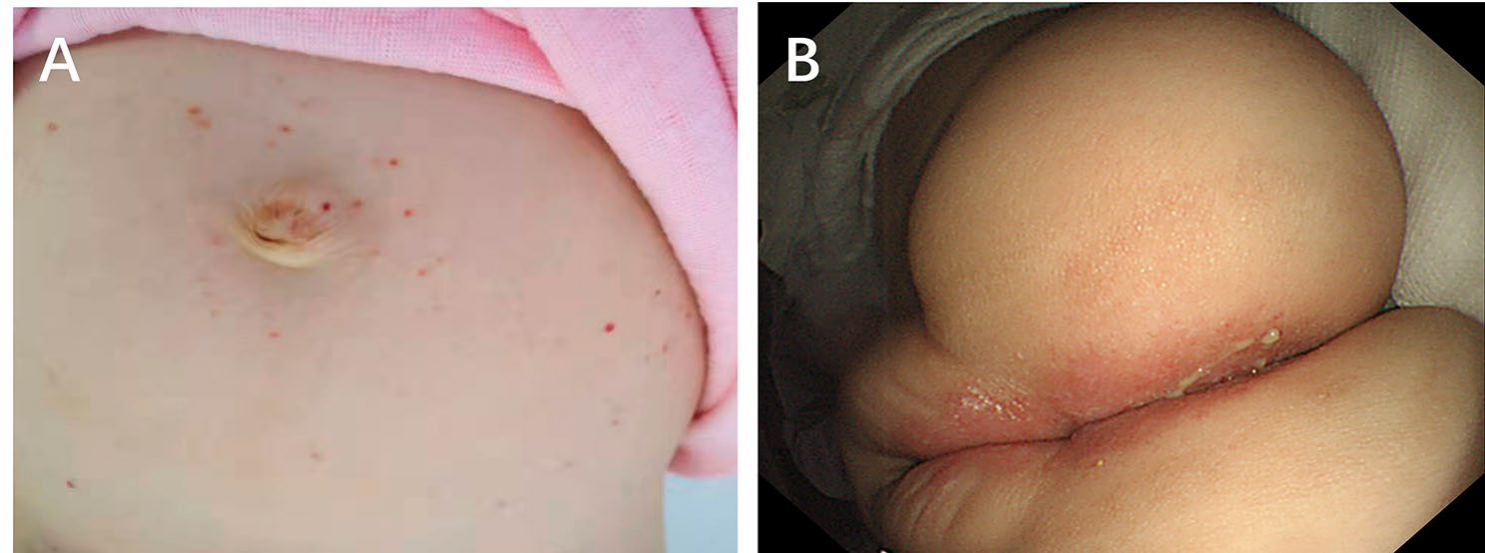
Physical examination of the first patient. (**A**) Skin macules on the abdomen. (**B**) The perianal skin was inflamed with white pus

On admission, her weight was 10 kg (WHO Z score − 0.32), and her height was 83 cm (WHO Z score 0.51). Upon physical examination, she was pale and had a few erythaematous lesions on her abdomen (Fig. 1A) and pitting oedema on her feet. The perianal skin inflamed with white pus (Fig. 1B). No icterus, lymphadenopathy, or organomegaly was noted. Laboratory indices revealed anaemia, as indicated by a haemoglobin level of 87 g/L, and hypoproteinaemia, as indicated by an albumin concentration of 15.7 g/L. Her stool sample for occult blood analysis was positive, and her WBC was 0–2/HP. The infection-related indicators, C-reactive protein (CRP), white blood cell (WBC) count, procalcitonin level and platelet count, were normal. The immunoglobulin level, liver and renal function, lactate dehydrogenase level, erythrocyte sedimentation rate (ESR), and T-SPOT were normal. Serology for Epstein–Barr virus (EBV), cytomegalovirus (CMV), hepatitis B, hepatitis C, and human immunodeficiency virus (HIV) was negative, as were tests for food allergies, *C. difficile*, and stool culture. Chest X-ray and abdominal computed tomography (CT), pituitary magnetic resonance imaging (MRI), lymph node ultrasound tests and bone marrow examination were negative. The main symptoms and abnormal indicators are shown (Table 1).

**Table 1:** The investigations conducted in both patients

		Patient 1	Patient 2
Characteristics	Age, m	18	14
	Gender	Female	Female
Main syndromes		Diarrhoea; intermittent fever	Diarrhoea; intermittent fever
Physical examination		A few erythaematous lesions on abdomen	Many erythaematous lesions on abdomen
Laboratory indices	Main abnormal index	Haemoglobin: 87 g/L Albumin: 15.7 g/L	Haemoglobin: 92 g/L Albumin: 22 g/L
	Stool tests	Occult blood +, WBC 0–2/HP	Occult blood +, WBC 0–1/HP
	Routine urinalysis	Negative	Negative
	Serum infection indicators	Negative	WBC count 19.84 × 10^9^/L, Neutrophils 6.5 × 10^9^/L Procalcitonin 1.77 ng/mL,
	Pathogen (T-SPOT, EBV, CMV, HIV, *C. difficile*)	Negative	Negative
	liver enzymes	Negative	Negative
	renal function	Negative	Negative
Pituitary MRI		Negative	Negative
Bone marrow examination		Negative	S100 and CyclinD1 histiocytes infiltrated
Endoscopy	Gastroenteroscopy	Erosion and narrowing of the duodenum, with white-tipped villi resembling snowflakes in small intestinal mucosa	Rough musosa in small intestine and atrophied villi
	Colonoscopy	Multiple haemorrhagic spots and oedema in the colonic mucosa	Oedema in the colonic mucosa
Biopsy		Langerhans cells, and immunohistochemical assessment revealed CD1a and Langerin	Langerhans cells, and immunohistochemical assessment revealed S100, Vim, CD1a, CyclinD1 and Langerin
Positron emission tomography -CT		Bone and pneumonia were involved	Liver and bone marrow were involved

*CRP* C-reactive protein, *WBC* white blood cell, *EBV* Epstein–Barr virus, *CMV* cytomegalovirus, *HIV* human immunodeficiency virus, *C. difficile* Clostridium difficile, *MRI* magnetic resonance imaging

**Fig. 2:**
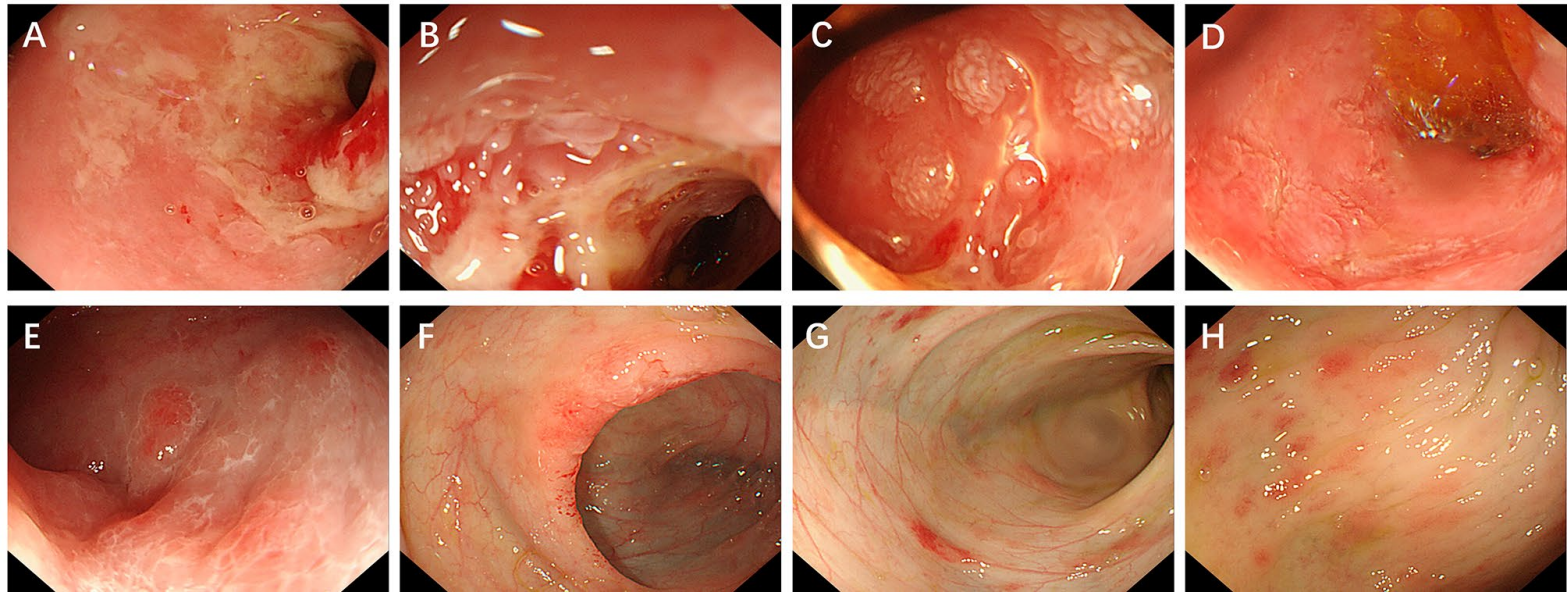
Endoscopic images for the first patient. (**A, B**) Erosion and ulcerations of the mucosa, as well as narrowing of the duodenum, through which a 5.8-mm diameter endoscope was able to pass. (**C–E**) Multiple, white-tipped villi resembling snowflakes in the small intestinal mucosa (the descending part of the duodenum to the beginning of the jejunum and distal ileum). (**F**) Mucosal swelling and erosions of the mucosa of the ileocecal valve. (**G, H**) Multiple haemorrhagic spots and oedema were observed in the colonic mucosa, extending from the ascending colon

Due to intermittent low-grade fever, ceftazidime and metronidazole were given to prevent infection. Treatment with omeprazole and octreotide inhibited the secretion of digestive fluid to alleviate diarrhoea. Regular albumin supplementation relieved anasarca, and parenteral nutrition improved the patients’ nutritional statuses. Gastrointestinal endoscopies were performed after admission. Upper endoscopy revealed erosion and narrowing of the duodenum, through which a 5.8-mm diameter endoscope was able to pass. There are multiple, white-tipped villi resembling snowflakes in the small intestinal mucosa (the descending part of the duodenum to the beginning of the jejunum and distal ileum). Colonoscopy revealed multiple haemorrhagic spots and oedema in the colonic mucosa (Fig. 2). Biopsy specimens obtained during upper endoscopy and colonoscopy were sent for pathological evaluation. Initial pathologic examination revealed that inflammatory bowel disease could not be excluded. The preliminary diagnosis was inflammatory bowel disease with lymphatic dilation of the small intestine.

**Fig. 3:**
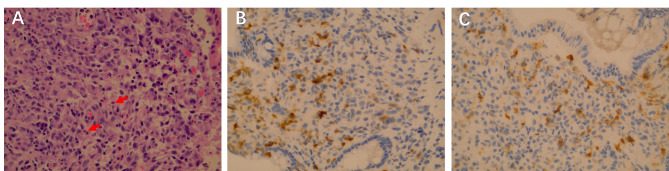
Pathologic examination (**A**) Images of the colonic mucosa stained with haematoxylin and eosin (H&E) revealed the presence of mononuclear cells with abundant cytoplasm and convoluted nuclei (×400). (**B**) Immunohistochemically stained colon sample infiltrated with Langerin on histiocytes (×400). (**C**) Immunohistochemically stained colon sample with CD1a expression on histiocytes (×400)

**Fig. 4:**
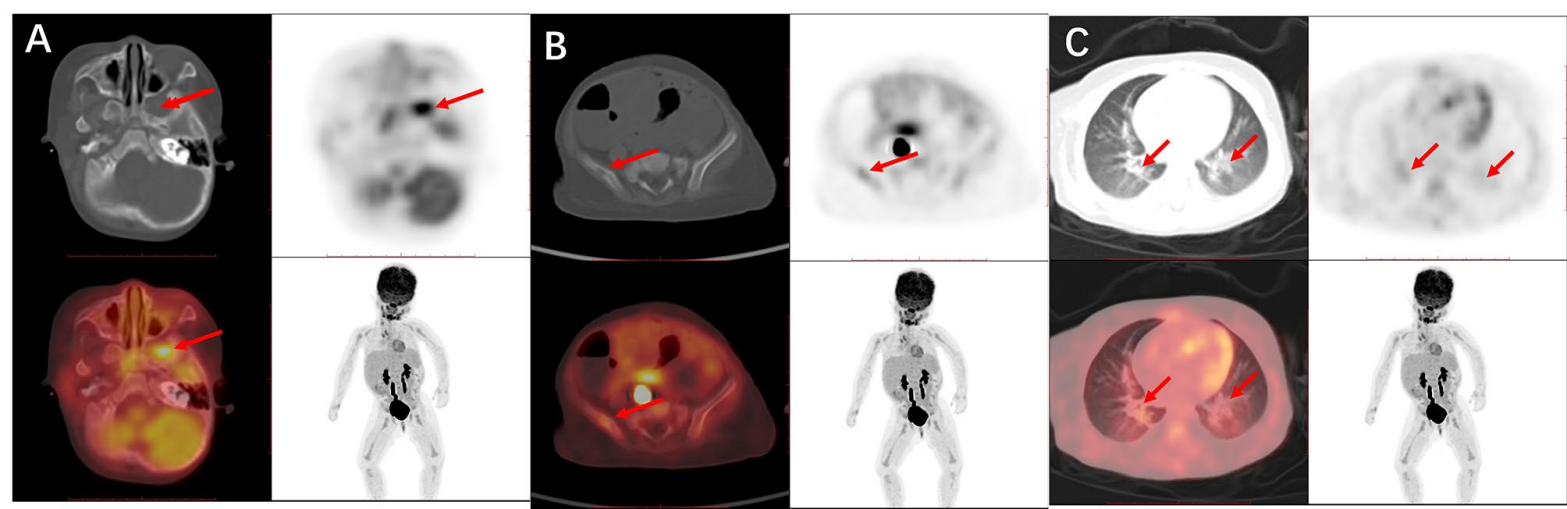
Positron emission tomography (PET)-CT image showing osteolytic lesions in the great wing of the sphenoid bone (**A**), the iliac bone (**B**) and pneumonia-like manifestations (**C**)

Because of repeated eruptions on the abdomen, a biopsy was performed for pathologic identification of allergic purpura. Pathologic examination suggested LCH. Gastrointestinal mucosal tissue was subjected to immunohistochemical examination and stained positive for CD1a and Langerin, as well as for the BRAF^V600E^ mutation (Fig. 3). Positron emission tomography (PET)-CT showed osteolytic lesions in the great wing of the sphenoid bone, suggesting that the condition of the iliac bone and pneumonia-like manifestations were possibly exacerbated by LCH (Fig. 4), although the bone marrow examination showed no abnormalities. Based on GIT findings and skin, bone and lung involvement, the patient was diagnosed with LCH (multiple system involvement). The patient subsequently received chemotherapy with Vincristine 1.5 mg/m^2^ d1 once every week, daily oral prednisolone (40 mg/m^2)^ and cytarabine (100 mg/m^2)^ d1-4 once every two weeks for the first 6 weeks, and 6-mercaptopurine (50 mg/m^2^/d) was added according to the SCMC-LCH-2018 Group 1 protocol (Table 2). During the subsequent 2-year follow-up period, the patient had no recurrence of diarrhoea or hypoalbuminaemia.

**Table 2:** The comparison of treatments for high-risk patients with LCH

	Initial treatment course I	Initial treatment course II	Continuation treatment
SCMC-LCH-2018 Group1	(W1-W6) Pred 40 mg/m^2^ × 4w, taper 2w VCR 1.5mg/m^2^ iv dl qw Ara-C 100mg/m^2^ iv/IH d1-4 q2w(w1,3,5)	(W7-W22) Pred 40 mg/m^2^ d1-5 q3w VCR 1.5mg/m^2^ iv dl qw Ara-C 100mg/m^2^ iv/IH d1-4 q3w 6-MP 50mg/m^2^/d, po, qn	(W25-W52) Pred 40mg/m^2^ d1-5 q3w VCR 1.5/m^2^ iv d1 q3w Ara-C 100mg/m^2^ iv/H d1-4 q6w x3 times (w25,31.37) 6-MP 50mg/m^2^/d, po, qn
CCHG-LCH-2019	(W1-W6) Pred 40 mg/m^2^ × 4w, taper 2w VCR 0.05 kg/m^2^ iv day1 of w1,2,3,4,5,6	(W7-W30) Pred 40mg/m^2^ d1-5 q3w VCR 0.05 kg/m^2^ iv d1 q3w Ara-C 150mg/m^2^ iv/IH d1-5 q3w 6-MP 50mg/m^2^/d, po, qn	(W31-W 52) Pred 40mg/m^2^ d1-5 q3w VCR 0.05 kg/m^2^ iv d1 q3w 6-MP 50mg/m^2^/d, po, qn
Histiocyte Society LCH-III trial	(W1-W6) Pred 40 mg/m^2^ × 4w, taper 2w VCR 6mg/m^2^ iv dl qw	(W7-W12) Pred 40 mg/m^2^/d, d1-3 qw VCR 6mg/m^2^ iv dl qw	(W13-W52) Pred 40 mg/m^2^/d d1-5 q3w VCR 6 mg/m^2^/d iv q3w 6-MP 50 mg/m^2^/d for 12 months

### Patient 2

A 14-month-old girl with a 4-month history of diarrhoea and a 1-month history of generalized anasarca and intermittent fever was admitted to our hospital. She was found to have recurrent hypoalbuminaemia.

**Fig. 5:**
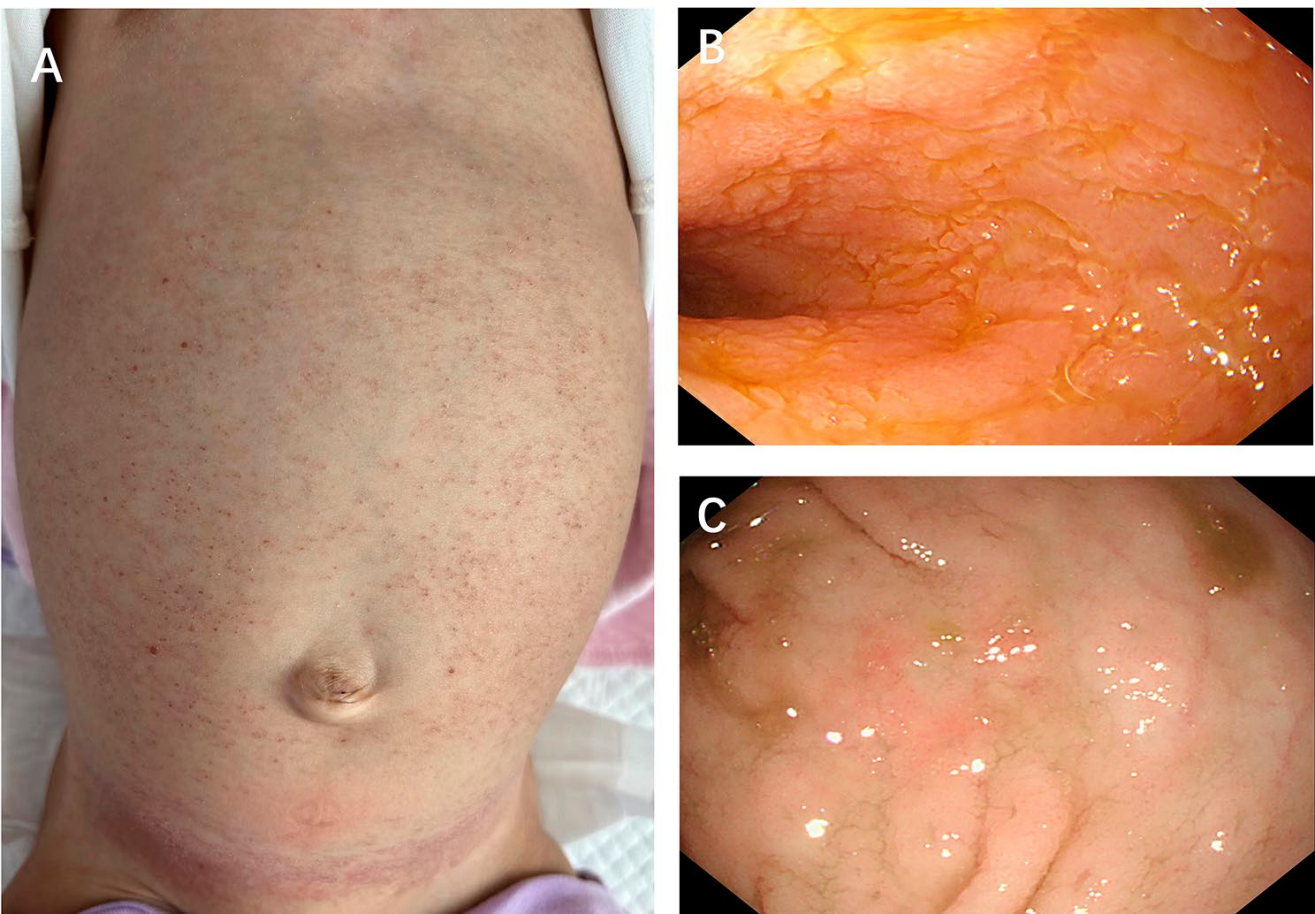
Rash and endoscopic images of the second patient. (**A**) The rash on the abdomen. (**B**) The mucosa was rough, and villi were atrophied in the descending part of the duodenum. (**B**) The colonic mucosa showed oedema and erosion

On admission, her weight was 8.6 kg (WHO Z score − 0.78), and her height was 75 cm (WHO Z score − 0.65). Upon physical examination, she was pale, with abdominal distention and many erythaematous lesions on her abdomen (Fig. 5A). Her perianal skin was normal. No icterus, lymphadenopathy, or organomegaly was noted.

Laboratory indices revealed anaemia, as indicated by a haemoglobin level of 92 g/L, and hypoproteinaemia, as indicated by an albumin level of 22 g/L. Her stool sample for occult blood analysis was positive, and her WBC was 0–1/HP. The following is the results of the assessment of infection-related indicators: white blood cells (WBC) count 19.84 × 10^9^/L, neutrophils 6.5 × 10^9^/L, C-reactive protein (CRP), procalcitonin 1.77 ng/mL and faecal calprotectin > 1800 ug/g. Urine output, immunoglobulin levels, liver enzymes, renal function, ESR and T-SPOT were normal. Serology for EBV, CMV, hepatitis-B, hepatitis-C, *C. difficile* and HIV was negative. Her chest X-ray and abdominal computed tomography (CT), pituitary magnetic resonance imaging (MRI) and lymph node ultrasound results were negative. The main symptoms and abnormal indicators are shown (Table 1).

The patient had intermittent fever after admission, and antibiotic treatment did not decrease her temperature, frequency of defecation or infection indices. Hypoalbuminaemia improved after albumin supplementation but recurred without it. We performed skin biopsy, gastroenteroscopy and PEC-CT. Gastroenteroscopy revealed that the small intestine was rough and that its villi were atrophied and that the colonic mucosa was oedematous (Fig. 5). Biopsy of both the gastrointestinal tissue and the rash revealed Langerhans cells, and immunohistochemical assessment revealed S100, Vim, CD1a, CyclinD1 and Langerin staining, with a BRAF V600E mutation. PEC-CT showed that her liver and bone marrow were involved. Then, bone marrow aspiration was performed to show the extent of S100 and CyclinD1 histiocytes infiltrated the tissues, which is characteristic of LCH. The patient was subsequently diagnosed with LCH (with multiple system involvement). She received weekly chemotherapy with Vincristine 0.05 mg/kg d1 once every week and daily oral prednisolone 40 mg/m^2^ for 6 weeks. Cytarabine 100 mg/m^2^ was added after 6 weeks, according to the CCHG-LCH-2019 protocol (Table 2). After she received chemotherapy, her symptoms of diarrhoea were significantly relieved.

## Discussion and conclusions

Both of our two patients less than 2 years old. Upper GIT endoscopy revealed scattered superficial erosions, haemorrhagic ulcerations or villous atrophy from the duodenum to the beginning of the jejunum. Colonoscopy revealed that the entire colonic mucosa was oedematous with multiple erythaematous lesions, and ulcers were also occasionally observed in the rectal mucosa. The time from symptom onset to diagnosis was approximately five months. The symptoms in both patients improved significantly after receiving chemotherapy according to the SCMC-LCH-2018 Group 1 and CCHG-LCH-2019 protocols.

GIT involvement in LCH is rare. Due to its rarity and lack of clinical report, 4 patients were reportedly misdiagnosed with cow protein allergy, neonatal enterocolitis or inflammatory bowel disease [2–5]. The reported incidence of LCH ranges from 2.6 to 8.9 cases per million children younger than 15 years per year [6]. In the other 6 reports in the literature in which GIT symptoms were the initial manifestation, all 7 patients were younger than 2 years [2, 4, 5, 7–9]. It seems that GIT-LCH was common in children aged < 2 years at the time of diagnosis.

The clinical symptoms of LCH-GIT include nausea, vomiting, abdominal pain, diarrhoea, haematochezia, constipation, intestinal obstruction, intussusception, and intestinal perforation [10, 11]. GIT symptoms lack specificity and are difficult to identify, and it is difficult to diagnose LCH without lesions in other systems (i.e., the lung or bones) at initial presentation. The main gastrointestinal symptoms in our two patients were refractory diarrhoea and protein-losing enteropathy. Among the above 7 patients, the initial GIT symptoms were diarrhoea in 5 patients, abdominal distension in 1 patient, and vomiting in 1 patient, while hypoalbuminaemia was reported in 6 patients [2, 4, 5, 7–9].

GIT endoscopy is important for identifying the cause of chronic diarrhoea. As GIT symptoms in LCH patients are rare, few cases of endoscopic manifestations have been reported. Combining the endoscopic findings of our two patients with those of other case reports, patchy erythema of the colorectal mucosa and narrowness and erosion of the distal duodenum might be suggestive manifestations of GIT involvement in LCH on endoscopic examination [5, 7, 8]. However, it is still difficult to directly distinguish LCH from other diseases, such as inflammatory bowel disease, by endoscopic manifestations.

Pathologic examination is the gold standard for definitive diagnosis of LCH. Typical LCH lesions show large cells, pale cytoplasm, and reniform nuclei on haematoxylin and eosin staining. LCH lesions are granulomatous lesions consisting of pathologic “Langerhans cells” (LCs), lymphocytes (primarily T cells), eosinophils, and macrophages [12]. LCH lesion LCs express CD1a, S100 and langerin surface markers according to immunohistochemical examination, and alternative BRAF mutations have also been described [1, 12]. As the diagnosis of LCH is based on histology, experienced clinicians should guide pathologists in identifying the manifestations of LCH and perform corresponding immunohistochemical staining tests.

Rashage and bone presentation were the main abnormalities in addition to GIT symptoms in our patients. The rash was easy to ignore initially due to a lack of clinical experience. Rash was found in 5 cases with GIT-LCH; all of these cases involved the trunk and presented with scattered red papules, but only two of them showed rash [2, 4, 5, 7–9]. If this typical lesion can be recognized, it may be possible to perform a skin biopsy for early definitive diagnosis.

Bone involvement is also characteristic of LCH and can present as either single or multiple osteolytic lesions, including those of the skull, mandible, spine, or long bones [10]. In our case, bone-related X-rays, CT and MRI were negative, but PET-CT showed osteolytic lesions in the bones or involvement in the liver and bone marrow. Therefore, PEC-CT is sensitive and necessary when considering the diagnosis of LCH. It is beneficial to locate the other organs involved in LCH to divide patients into single- or multiple-system groups precisely for further treatment.

Due to its rarity and the lack of known cases, the initial presentation of LCH-GIT is easy to misdiagnose. When refractory diarrhoea is accompanied by recurrent hypoalbuminaemia, especially when accompanied by abdominal rash, LCH should be considered. Endoscopic and pathologic examinations are essential for definitive diagnoses, while PET-CT is necessary to determine the involvement of other organs. All examination methods are conducive to treatment.

## Data Availability

The datasets used and analysed during the current study are available from the corresponding author upon reasonable request.
